# The Glutamatergic Neurons in the Spinal Cord of the Sea Lamprey: An *In Situ* Hybridization and Immunohistochemical Study

**DOI:** 10.1371/journal.pone.0047898

**Published:** 2012-10-22

**Authors:** Blanca Fernández-López, Verona Villar-Cerviño, Silvia M. Valle-Maroto, Antón Barreiro-Iglesias, Ramón Anadón, María Celina Rodicio

**Affiliations:** Department of Cell Biology and Ecology, University of Santiago de Compostela, Santiago de Compostela, Spain; Emory University, United States of America

## Abstract

Glutamate is the main excitatory neurotransmitter involved in spinal cord circuits in vertebrates, but in most groups the distribution of glutamatergic spinal neurons is still unknown. Lampreys have been extensively used as a model to investigate the neuronal circuits underlying locomotion. Glutamatergic circuits have been characterized on the basis of the excitatory responses elicited in postsynaptic neurons. However, the presence of glutamatergic neurochemical markers in spinal neurons has not been investigated. In this study, we report for the first time the expression of a vesicular glutamate transporter (VGLUT) in the spinal cord of the sea lamprey. We also study the distribution of glutamate in perikarya and fibers. The largest glutamatergic neurons found were the dorsal cells and caudal giant cells. Two additional VGLUT-positive gray matter populations, one dorsomedial consisting of small cells and another one lateral consisting of small and large cells were observed. Some cerebrospinal fluid-contacting cells also expressed VGLUT. In the white matter, some edge cells and some cells associated with giant axons (Müller and Mauthner axons) and the dorsolateral funiculus expressed VGLUT. Large lateral cells and the cells associated with reticulospinal axons are in a key position to receive descending inputs involved in the control of locomotion. We also compared the distribution of glutamate immunoreactivity with that of γ-aminobutyric acid (GABA) and glycine. Colocalization of glutamate and GABA or glycine was observed in some small spinal cells. These results confirm the glutamatergic nature of various neuronal populations, and reveal new small-celled glutamatergic populations, predicting that some glutamatergic neurons would exert complex actions on postsynaptic neurons.

## Introduction

Since the 1970s, glutamate has been recognized as the major excitatory neurotransmitter in the central nervous system of vertebrates [1]–[3]. It acts on several types of glutamate receptors: three groups of ionotropic receptors and three groups of metabotropic receptors (mGluR) [4], [5]. Glutamate is also involved in important processes in the developing brain such as neuronal differentiation and migration [4], [6], [7]. Glutamate probably exerts influence on neuronal responses to some basic guidance molecules [8]. As a neurotransmitter, glutamate plays a major role in the processing and transmission of sensory information in the spinal cord [9], [10] and in the spinal circuits involved in locomotion [11], [12]. In mammals, all primary afferents to the spinal cord use glutamate as their major fast transmitter [10], [13]. Glutamate is introduced from the extracellular medium to the neuron cytoplasm by an excitatory amino acid transporter (EAAT; common for glutamate and aspartate) and then it is transported into the synaptic vesicles by vesicular glutamate transporters (VGLUTs) specific for this amino acid. Three VGLUTs have been identified in mammals to date (VGLUT1, VGLUT2 and VGLUT3) [14]. Early anatomical studies of the glutamatergic system in mammals depended on the use of antibodies raised against glutamate-protein conjugates [15], [16]. The use of these antibodies to characterize glutamatergic neurons was often considered problematic, because glutamate is a metabolic molecule found in all cells and because staining of perikarya was somewhat inconsistent [17]. Accordingly, some authors have considered that there were no reliable immunocytochemical markers for the cell bodies of glutamatergic neurons in mammals. In this way, glutamatergic cells were often defined by negative criteria (those cells that were not immunoreactive for GABA and glycine) [10]. The new studies with in situ hybridization for vesicular glutamate transporters allowed identifying unequivocally glutamatergic neuronal perikarya, whereas immunohistochemistry with antibodies raised against these transporters have been useful to identify axons of glutamatergic cells although they fail to stain the perikaryon [10], [13]. Studies of VGLUT distribution in the rat lumbar spinal cord indicate that the proportion of glutamatergic neurons clearly exceeds the half of the total number of neurons in all laminae [13]. The morphology of glutamatergic neurons of lamina I and II of the rat dorsal horn has been investigated in detail using combined physiological and immunohistochemical methods to characterize vesicular glutamate transporters in axons of neurobiotin-injected single cells [10], [18], [19].

The spinal cord has an intrinsic circuitry that controls locomotion generating a coordinated rhythmic output; this circuitry is known as the spinal cord central pattern generator (CPG) [20]. The excitatory interneurons play an important role in the rhythm generation (reviewed by [20]–[22]). Lampreys have been used for many years as a model to identify the neuronal circuits involved in the control of locomotion (see [20], [23]). Actually, lampreys are the vertebrates for which more detailed knowledge about the spinal neuronal network is available, mostly based on electrophysiological and anatomical studies (see [24], [25]). Distribution of glutamatergic neurons in the spinal cord has been studied in a few animal species by immunohistochemistry with antibodies raised against glutamate-protein conjugates or antibodies raised against vesicular glutamate transporters (VGLUTs), and by *in situ* hybridization with probes for VGLUTs. The majority of these later studies were made in adult mammals (rat: [2], [13], [26]; mouse: [3]) and in developing zebrafish [12]. The CPG is composed by several types of interneurons that control the discharge of motoneurons (reviewed by [20], [27]). Excitatory interneurons excite motoneurons and other interneurons, inhibitory glycinergic interneurons ensure alternate left-right side segmental activation by inhibiting all neuronal types on the contralateral side. In addition to interneurons, lamprey edge cells (a class of intraspinal stretch receptor neurons) provide sensory feedback to the rhythm-generating network. Some edge cells excite ipsilateral neurons, whereas others inhibit contralateral neurons [28].

Lampreys belong to the Agnathans, the oldest group of extant vertebrates, and thus have a great value for deciphering the early evolution of neurochemically-defined systems. Several studies have revealed the organization of glycinergic [29]–[31] and GABAergic [32]–[36] cells in the lamprey spinal cord. However, the distribution of glutamatergic cells in the spinal cord has been studied only with glutamate immunohistochemistry either in synapses at ultrastructural level [37] or in some cell perikarya of commissural neurons [38]. To the best of our knowledge, there has been no report in non-mammalian adult vertebrates of the expression of VGLUTs in the spinal cord, or comprehensive studies of spinal glutamatergic populations using glutamate immunohistochemistry. The recent cloning of a cDNA coding for a lamprey vesicular glutamate transporter (VGLUT) [39] affords an alternative tool to assess the glutamatergic character of lamprey neurons, as reported in the brain [40], [41].

Over the past two decades, evidence indicating that transmission by multiple messengers released by single neurons was the norm rather than the exception has been accumulated [42]–[46]. The functional implications of neurotransmitter co-release are not clear but it likely plays an important role in the maturation and refinement of synapses, in precision of motor activity, in the homeostatic opposition to hyperexcitability during seizures [42] or possibly in reducing the metabolic cost and errors of signaling [43]. Glutamate has been shown to colocalize with other neurotransmitters in some neurons. Colocalization of glutamate and GABA immunoreactivity has been reported in several regions of the central nervous system of mammals (for review see [47], [48]). Colocalization of glutamate and glycine immunoreactivity has also been observed in some central neurons of mammals and amphibians [49]–[51]. With regard to lampreys, several reports indicate that they are good to study distribution and colocalization of amino acid neurotransmitters at cellular level [31], [40], [45], [46], [52]–[56]. Knowledge of the colocalization of glutamate and GABA or glycine in cells of the lamprey spinal cord may contribute to a better understanding of their roles in the locomotor circuits.

The main aim of this study was to characterize the glutamatergic neuronal populations of the spinal cord of the sea lamprey, *Petromyzon marinus*. For this goal, we investigated the expression of VGLUT in neuronal perikarya to assess the glutamatergic character of cells. We also compared, by means of double immunofluorescence and confocal microscopy, the distribution of glutamate with that of GABA and glycine, the major inhibitory neurotransmitters in the central nervous system. This study determined the distribution of several populations of glutamatergic neurons along the spinal cord. Moreover, the results indicate that some glutamate-ir cells, also show GABA or glycine immunoreactivity. These results were discussed in a comparative and functional context.

## Results

### VGLUT Expression and Glutamate Immunoreactivity in Neuronal Populations in the Lamprey Spinal Cord

In lampreys, the spinal cord is flattened and the gray matter forms paired “wings” or “horns” that extend laterally. The medial region corresponds to the dorsal horn of the spinal cord in jawed vertebrates in terms of embryonic origin and the lateral region corresponds to the ventral horns (see [34]). VGLUT-expressing and glutamate-immunoreactive neurons were distributed in the gray and white matter of the spinal cord. Interestingly, similar neuronal populations were observed with both VGLUT *in situ* hybridization and glutamate immunohistochemistry. The number of labeled cells per section with both methods is shown in Table 1. The means and standard deviations founded suggest that the two methods are revealing the same neuronal populations. Important differences among individuals in the number of neurons in the lamprey spinal cord has been previously reported [57]. Since the possibility that some glutamate-ir neurons do not actually correspond with VGLUT positive cells cannot be ruled out, in the following we refer to glutamate-ir neurons as putative glutamatergic cells. Accordingly, the description of the populations is based on *in situ* hybridization experiments whereas a more detailed description of the size, morphology and processes is based on immunohistochemistry studies.

**Table 1:** Comparison between numbers of glutamate and VGLUT positive cells per section.

	GRAY MATTER						WHITE MATTER			
	DORSAL CELLS	DORSAL POPULATION		LATERAL POPULATION		CSFc CELLS	EDGE CELLS	MAC	MTAC	MDLAC
		DM	DL	LARGE	SMALL					
Glutamate	0.05±0.22	0.92±1.05	0.63±0.82	0.88±0.84	7.28±2.97	1.32±1.18	0.22±0.41	0.28±0.49	0.05±0.22	0.07±0.25
VGLUT	0.25±0.44	0.60±0.67	0.88±0.60	0.92±0.79	9.07±2.35	1.68±0.75	0.48±0.51	0.30±0.46	0.12±0.33	0.20±0.41

DM (Dorsomedial); DL (Dorsolateral); MAC (Müller axons associated cells);

MDLAC (Medium-sized dorsolateral axons associated cells); MTAC (Mauthner axons associated cells).

The size of larval cells is much different from the size of cells in adults. For descriptive purposes, larval neurons with mean minor cell diameter less than 10 µm are described as small, cells between 10 and 25 µm as large, and those larger than 25 µm as giant. In adult animals, cells less than 25 µm in diameter are referred as small, cells between 25 and 50 µm as large, and those clearly exceeding 50 µm in diameter as giant.

**Figure 1:**
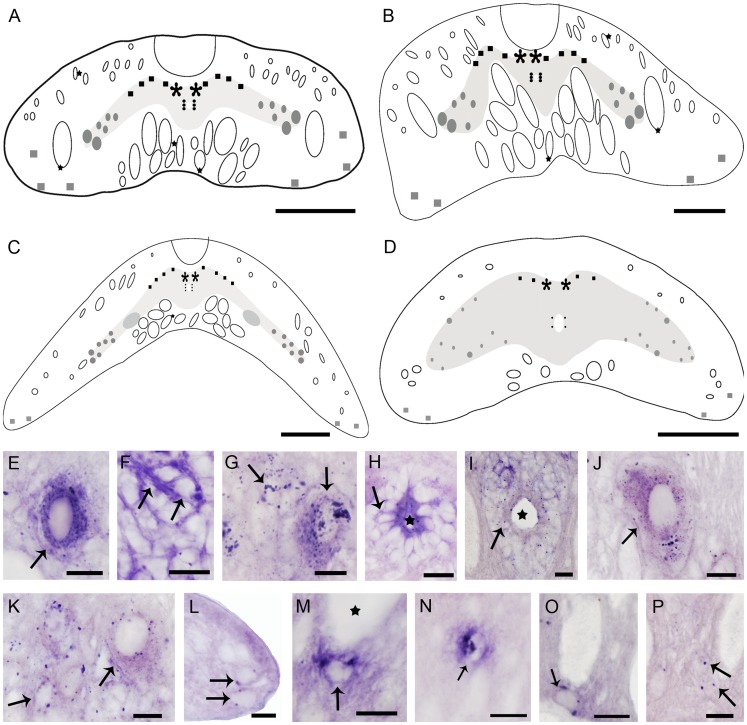
Lamprey vesicular glutamate transporter (VGLUT) expression in transverse sections of the spinal cord. A: Schematic drawing showing the distribution of glutamatergic cell types in rostral spinal levels: primary sensory neurons or dorsal cells (black asterisks); dorsal interneurons (black squares); cells of the lateral population (gray circles); cerebrospinal fluid-contacting cells (CSFc) cells (black circles); edge cells (gray squares); cells associated with reticulospinal and Mauthner axons (black stars).**B:** Schematic drawing showing the distribution of the major VGLUT-positive cell types in middle levels of the larval spinal cord. Cell type symbols as in Figure 1A. **C:** Schematic drawing showing the VGLUT-positive neuronal populations in the dorsal fin region; same symbols as in Figure 1A. **D:** Schematic drawing showing the VGLUT-positive neuronal populations in the caudal fin region; same symbols as in Figure 1A. **E–P:** High magnification photomicrographs of larva and adult showing details of VGLUT-positive cells (arrows) in the spinal gray (E–K) and white matter (L–P). **E:** Dorsal cell. **F:** Dorsal interneurons. **G:** Small and large lateral interneurons. **H, I:** CSFc cells of a larva (H) and an adult (I), star indicates the central canal. **J:** VGLUT-positive giant cell. **K:** Small and large lateral neurons. **L:** VGLUT-positive edge cells. **M:** Glutamatergic neuron situated ventrally to the Mauthner axon (star). **N:** Glutamatergic cell associated with medium-sized axons of the lateral column. **O:** VGLUT-positive cells situated ventrally to Müller axons. **P:** VGLUT-positive neurons situated among the Müller axons. Dorsal is at the top. Lateral is on the left except in G, K, L and O, in which lateral is on the right. F, I, J, K and P correspond to adult individuals. E, G, K, M, N and O correspond to the rostral spinal cord; I and P correspond to the middle spinal cord; H, L and J correspond to the dorsal fin level; F correspond to the caudal fin level. Scale bars = 100 µm (A); 50 µm (B, C, D); 10 µm (E, F, G, H, I, J, K, M, N, O, P); 5 µm (L).

The patterns of VGLUT expression and glutamate immunoreactivity in the spinal cord of larval (Fig. 1A–D) and adult sea lamprey are quite similar. A notable difference was the size of glutamatergic cells, which were two or three times larger in adults than in larvae (as example, Table 2 shows the sizes of gray matter glutamatergic interneurons). In our slices of the most caudal spinal cord (caudal fin region) of upstream migrating adults, the levels of glutamate immunoreactivity were low so it was not possible to measure the diameters of cells. However, VGLUT expression and glutamate immunoreactivity were clearly observed at this level of the spinal cord in downstream migrating adults (Fig. 1F). Moreover, a decrease in the cellular density occurred in the adult spinal cord, and the glutamate-ir cells appear more scattered than in larval lampreys, as observed with immunohistochemistry. It was easier to distinguish the cell morphology in larvae than in adults: accordingly, the descriptions were based in larval observations. As is stated in Material and Methods, the VGLUT expression in upstream migrating adults was not investigated.

**Table 2:** Cell sizes of main glutamatergic populations.

	SIZES OF CELLS (µm)					
	LARVA			ADULT		
SPINAL LEVEL	DI	SLP	LLP	DI	SLP	LLP
ROSTRAL	9.2±1.4	9.5±1.0	14.4±2.3	16.8±2.4	21.1±3.1	45.6±6.5
MEDIAL	9.7±1.7	9.6±1.6	16.1±3.0	15.2±1.8	16.4±2.2	26.9±5.2
CAUDAL	6.2±0.8	6.9±0.9	11.1±1.5	NOTDONE	NOTDONE	NOT DONE

DI (dorsal interneurons); LLP (large lateral population); SLP (small lateral population).

In general, the distribution of VGLUT expression and glutamate-ir were similar at all the levels of the spinal cord: rostral (Fig. 1A), middle (Fig. 1B), dorsal fin (Fig. 1C) and caudal fin (Fig. 1D), excepting for the presence of giant glutamatergic cells at the dorsal fin level, the absence of cells associated with medium-sized dorsolateral axons at the dorsal and caudal fin levels and the absence of cells associated with Müller axons at the caudal fin level. In addition, at the caudal fin level, the transverse area of the spinal cord was reduced with respect to the other regions, as also were the sizes of the cells (Table 2) and the diameter of the giant and medium-sized axons. (Fig. 1D).

**Figure 2:**
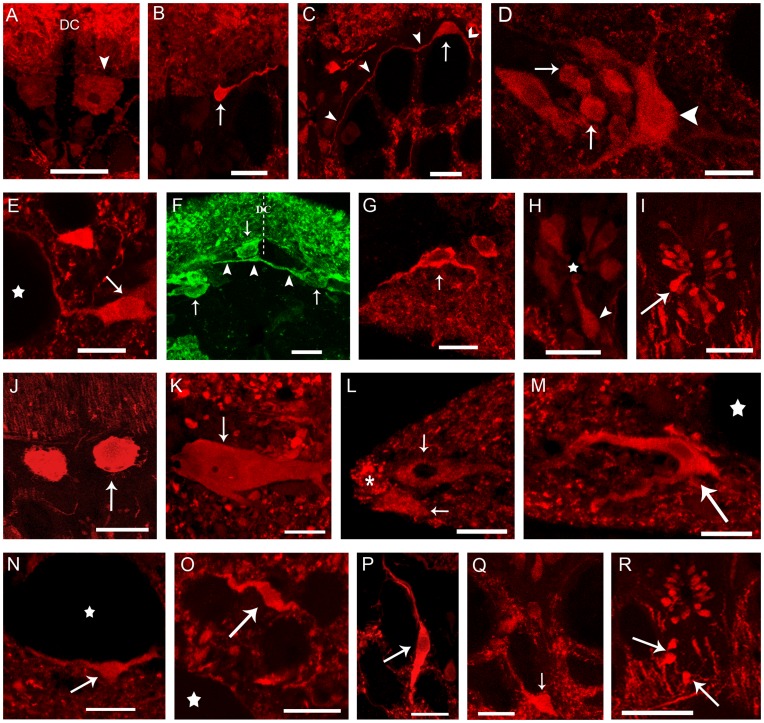
Glutamate immunoreactivity in transverse sections of the sea lamprey spinal cord. **A–R:** High magnification photomicrographs of larvae and adults showing details of glutamate-ir cells (arrows) and processes in the spinal gray matter at different rostro-caudal levels. **A:** Dorsal cells (arrowhead), DC indicate the dorsal column. **B, C:** Dorsal interneurons. **B:** Glutamate-ir cell with thick (dendrite) and thin (axon) processes directed to the dorsal column. **C:** Glutamate-ir bipolar cell showing ventrolateral (barbed arrowhead) and ventromedial (pointed arrowheads) processes. **D**: Glutamate-ir small (arrow) and large (arrowhead) neurons of the lateral gray. **E:** Lateral cell with a process surrounding the Mauthner axon (star). **F:** Glutamate-ir cells (arrows) of the dorsal population showing processes directed to the dorsal column (DC) and a dendrite (arrowheads) that crosses the midline (dashed line) dorsally. **G:** Detail of a glutamate-ir bipolar ventrolateral cell (arrow) of the caudal region of the cord. **H:** Cerebrospinal fluid-contacting (CSFc) cells (arrowhead). The star indicates the central canal. **I:** Glutamate-ir cerebrospinal fluid-contacting (CSFc) cells of an adult. **J:** Strong glutamate-ir primary sensory dorsal cells. **K:** Giant glutamate-ir cell. **L:** Glutamate-ir edge cells (arrows) and edge neuropil (asterisk). **M:** Glutamate-ir edge cell (arrow) laterally. The white star indicates the Mauthner axon. **N:** Positive neuron (arrow) situated ventrally to the Mauthner axon (white star). **O:** Glutamate-ir cell (arrow) associated with medium-sized axons of the lateral column (White star: Mauthner axon). **P, Q:** Glutamate-ir cells (arrows) situated among (P) and ventrally (Q) to Müller axons. **R:** Glutamate-ir cells situated in the ventromedial region of the white matter. Note also CSFc cells around the central canal. Note that the giant Müller axons (in C, R) are apparently glutamate-negative. In all figures dorsal is at the top. Lateral is on the left except in B, C, D, G and K, in which lateral is on the right. I, J, K and R correspond to adult individuals. A, B, C, E, H, J, M, N, O, P correspond to the rostral spinal cord; D, I, L, Q, R correspond to the middle spinal cord; K corresponds to the dorsal fin level; F, G correspond to the caudal fin level. Scale bars = 100 µm (I, J, R); 50 µm (K); 20 µm (A, B, C, D, E, L, M, N, O, P Q); 10 µm (F, G, H).

VGLUT expression was observed in cells of both the gray matter and the white matter (Fig. 1A–D). The reaction signal was located in the cytoplasm around the cell nucleus and occasionally in proximal dendrites, and showed a grainy appearance. There were large differences in intensity of signal (number of positive granules) among neurons. In the gray matter, prominent VGLUT expression was observed in primary sensory neurons (dorsal cells) situated in the mediodorsal region of the gray matter just adjacent to the central canal and below the dorsal column. In transverse sections the dorsal cells showed a characteristic rounded profile (Fig. 1E). In larvae, dorsal cells were faintly glutamate-ir (Fig. 2A), but they were strongly glutamate-ir in adults (Fig. 2J). Hybridization signal was observed in a population of small interneurons located in the dorsomedial gray and in a heterogeneous cell population with regard cell size and intensity of reaction situated in the lateral gray matter (Fig. 1F, G, K). The putative glutamatergic interneurons of the dorsal population showed strong glutamate immunostaining. According to their position, two types of cells were distinguished: dorsomedial and dorsolateral. Dorsomedial cells were located in the medial region of the dorsal population and under the fibers of the dorsal column. These putative glutamatergic cells had one or more processes of dendritic appearance coursing dorsally or dorsolaterally to the ipsilateral dorsal column or to the dorsolateral region near the dorsal column (Fig. 2B). Some of these cells had a dendritic process crossing the midline dorsal to the central canal, under the dorsal column (Fig. 2F). Glutamate-ir dorsolateral cells were observed only in the rostral spinal cord and in the rostral part of the middle spinal region. These glutamate-ir cells had a process coursing ventromedially, surrounding the medial longitudinal fascicle and another process directed to the ventrolateral area (Fig. 2C). In some of these cells, a dorsal branch arising from the ventrolateral process coursed to the dorsal column (not shown). The glutamate-ir interneurons of the lateral population also showed strong immunostaining. According to their size (Table 2), two VGLUT-positive cell subpopulations were distinguished: large and small (Fig. 1G, K). Large lateral cells were situated in the most lateral region of the gray matter. They were multipolar neurons, irregular in shape and with processes directed to the medial, ventral and lateral funiculi (Fig. 2D). Some of the processes coursing laterally reached the area adjacent to the Mauthner axon (Fig. 2E). Small lateral cells were located medially to the large lateral cells. They were multipolar cells, with irregular morphology. Lateral, ventral and medial processes were distinguished in some of these cells (Fig. 2 D). In the caudal fin region the density of cells of the lateral population was higher than the observed in the other regions of the cord and both, large and small cells occupied both lateral and ventrolateral positions (not shown). Bipolar ventrolateral glutamate-ir cells with processes directed laterally and ventromedially (Fig. 2 G) were observed in the caudal spinal cord.

Surrounding the central canal of the spinal cord, there were small VGLUT-positive and glutamate-ir bipolar neurons of cerebrospinal fluid-contacting (CSFc) type with perikarya located in the ependymal walls. The number of positive granules per cell was usually scant (Fig. 1H, I). In larvae, most of these cells were faintly glutamate-ir, although some of them presented strong glutamate immunoreactivity (Fig. 2H). In adults, they showed different degrees of glutamate immunoreactivity, although most of them were faint or moderately glutamate-ir (Fig. 2I, R). A short dendrite of these cells coursed to the central canal, ending as a club. Occasionally, a thin axonal process was observed arising from the opposite pole of the cell.

At dorsal fin levels of the spinal cord, giant VGLUT-positive (Fig. 1J) and glutamate-ir (Fig. 2K) cells [32.7 µm × 19.4 µm (larvae), 56.1 µm in width × 109.1 µm (adult) in major and minor diameters, respectively] were situated in a ventrolateral position of the gray matter. These multipolar neurons showed an oval perikaryon and processes directed medially, laterally and ventrally (Fig. 1J, 2K).

In the white matter, there were VGLUT signal (Fig. 1 L–P) and glutamate immunoreactivity (Fig. 2L–R) in numerous cells of the lateral, ventromedial and dorsolateral regions. In lateral and ventrolateral spinal regions, different white-matter VGLUT positive and glutamate-ir cells were distinguished according to their location and morphology. VGLUT-positive and glutamate-ir edge cells were situated in the ventrolateral (Figs. 1L, 2L) and lateral marginal (Fig. 2L, 2M) zones of the spinal cord. Some of them were bipolar cells and showed a long dendritic process that coursed laterally to the marginal neuropil and a process that coursed medially (not shown). Other edge cells were tripolar in appearance, with ventrolateral and lateral processes directed to the edge of the spinal cord, and a medial process (Fig. 2M). Some VGLUT positive and glutamate-ir cells were observed closely associated with the Mauthner axon (Figs. 1M, 2N), except at the caudal fin level. These cells were bipolar with processes surrounding it (Fig. 2N).

In the dorsolateral funiculus, occasional glutamate-ir and VGLUT expressing cells were located among the medium-sized axons of this column (Figs. 1N, 2O) at rostral and middle spinal cord levels. Some of these cells were bipolar neurons with processes coursing among dorsomedial axons (Fig. 2O). In the ventromedial funiculus, some glutamatergic cells were observed among the giant Müller axons. Some perikarya of similar glutamate-ir cells were almost vertical and spindle-shaped, with dorsal and ventral processes (Fig. 2P). Some other glutamatergic cells were situated under the giant axons (Fig. 1O) and similar glutamate-ir cells showed their processes coursing laterally and medially (Fig. 2Q, R).

### Colocalization of Glutamate and GABA or Glycine Immunoreactivities

Previous studies of the lamprey spinal cord reported the presence of glycine and/or GABA in some small-celled spinal populations, whereas immunoreactivity to these substances was absent in dorsal cells, motoneurons and large lateral cells, among others [31], [35], [38], which is confirmed with present observations. Here, colocalization with glutamate was only investigated in those populations exhibiting GABA and glycine.

**Figure 3:**
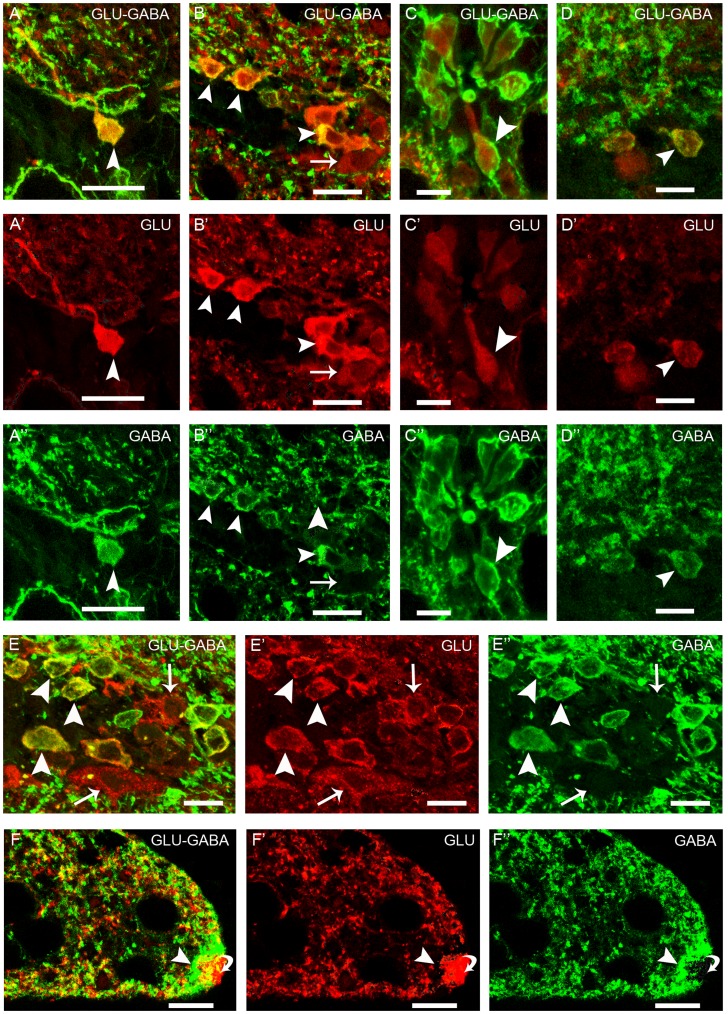
Glutamate and GABA colocalization in the lamprey spinal cord. **A–E”:** Confocal photomicrographs of transversal sections through the rostral, medial and caudal spinal cord of larvae showing double immunolabeled cells (arrowheads) for glutamate and GABA. Arrows point to single immunolabeled cells. **A–C”:** Rostral spinal cord. **D–D”:** Medial spinal cord. **E–E”:** Caudal spinal cord. **A–A”:** Dorsal glutamate-ir population. **B–B”:** Lateral glutamate-ir population. **C–C”:** Cerebrospinal fluid contacting cells. **D–D”:** Dorsal glutamate-ir population. **E–E”:** Lateral glutamate-ir population. **F–F”:** Confocal photomicrograph of a transverse section through the rostral spinal cord of a larva showing the glutamate-ir processes (curved arrow) surrounded by the GABA-ir fibers constituting the marginal neuropil (arrowhead). Dorsal is at the top. Lateral is on the right except for A–A”, in which lateral is on the left. A, B, C, D, E, F: Overlay; A’, B’, C’, D’, E’, F’: Glutamate; A”, B”, C”, D”, E”, F”: GABA. Scale bars = 20 µm (A–B”; F–F”); 10 µm (C–E”).

**Figure 4:**
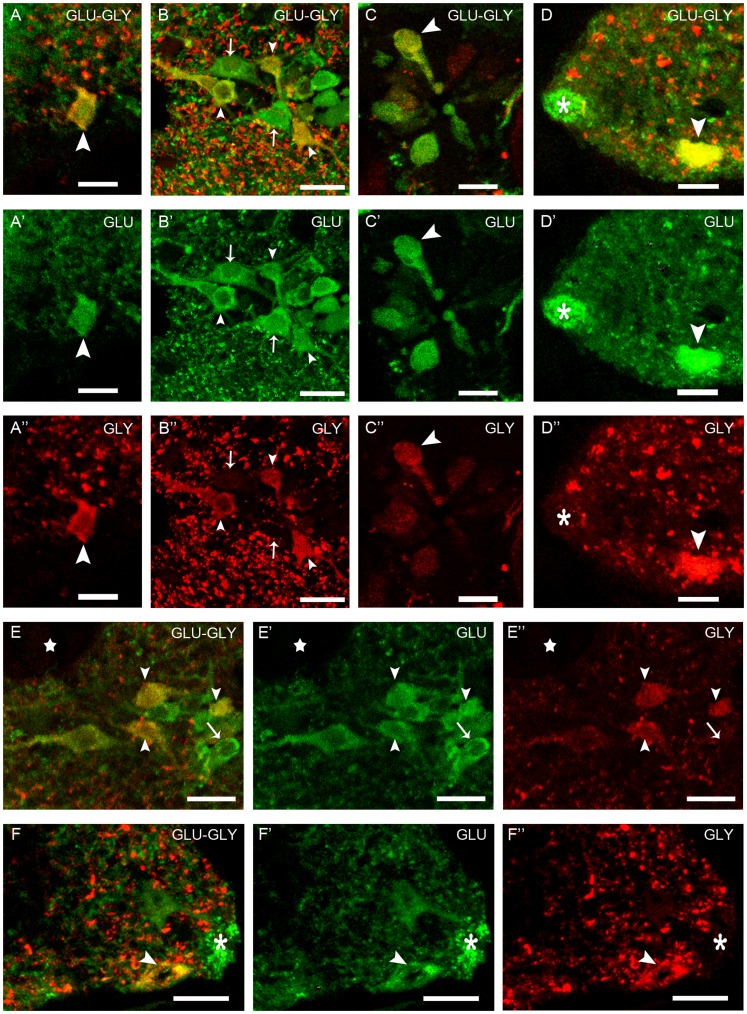
Glutamate and glycine colocalization in the lamprey spinal cord. **A–D”:** Rostral spinal cord. **E–E”:** Medial spinal cord. **F–F”:** Caudal spinal cord. **A–A”:** Dorsal glutamate-ir interneurons. **B–B”:** Lateral glutamate-ir population. **C–C”:** Cerebrospinal fluid contacting cells. **D–D”:** Edge cell and marginal neuropil. **E–E”:** Lateral glutamate-ir population. **F–F”:** Edge cell and marginal neuropil. In E–E” and F–F” note that the glutamate-ir marginal neuropil mostly lacks glycine immunoreactivity (asterisk). Arrowheads point to double immunolabeled cells and arrows point to single glutamate-ir cells. Dorsal is at the top. Lateral is on the left in B–B” and D–E”, and on the right in A–A” and F–F”. Scale bars = 10 µm (A–A’, C–F”), 20 µm (B–B”).

In the spinal cord, colocalization of glutamate and GABA immunoreactivities was observed in some medial glutamate-ir interneurons of the dorsal population (Fig. 3A–A”, D–D”), in small lateral cells (Fig. 3B–B”, E–E”) and in most of the CSFc cells (Fig. 3C–C”). Whereas these populations were observed in both larval and adult lampreys, the percentage of glutamate-ir cells showing colocalization with GABA varied among stages and spinal regions. Table 3 shows the percentages of double-labeled interneurons. They correspond to arbitrary lengths of spinal cord in which both dorsal and lateral glutamate-ir cells were counted. No colocalization with GABA immunoreactivity was observed in the glutamate-ir cells situated in the white matter or in other glutamate-ir cellular types of the gray matter. In the CSFc cells, the cellular distribution of glutamate and GABA immunoreactivities was not homogeneous along the cell. Thus, glutamate and GABA were colocalized in the apical dendrite including the terminal club and in the central region of the soma but only some of the CSFc cells showed glutamate immunoreactivity in the exit of the basal process. The periphery of the soma and the basal process in their whole length were only GABA-ir. Colocalization of glutamate and glycine was observed in in cells located in both the gray and the white matter. In the gray matter, some interneurons of the dorsal population (Fig. 4A–A”), some small lateral cells (Fig. 4B–B”, E–E”) and some CSFc cells (Fig. 4C–C”) showed glutamate and glycine colocalization. In these CSFc cells, the cellular distribution of immunolabeling was similar for both neurotransmitters, which is unlike to that reported for glutamate and GABA (see above). As reported above for GABA, regional differences in the degree of colocalization of glycine in glutamate-ir interneurons were also observed (Table 4), and the colocalization percentages obtained correspond to arbitrary lengths of spinal cord.

**Table 3:** Percentages of glutamate-ir cells showing GABA colocalization.

	GLU-GABA			
	LARVA		ADULT	
SPINAL LEVEL	DI	SLP	DI	SLP
ROSTRAL	38% (n = 138)	27% (n = 322)	22% (n = 268)	17% (n = 420)
MEDIAL	28% (n = 144)	21% (n = 371)	18% (n = 117)	12% (n = 276)
CAUDAL	18% (n = 243)	19% (n = 637)	NOT DONE	NOT DONE

DI (Dorsal interneurons); SLP (Small lateral population).

**Table 4:** Percentages of glutamate-ir cells showing glycine colocalization.

	GLU-GLY			
	LARVA		ADULT	
SPINAL LEVEL	DI	SLP	DI	SLP
ROSTRAL	19% (n = 172)	24% (n = 335)	20% (n = 184)	19% (n = 297)
MEDIAL	23% (n = 65)	17% (n = 193)	14% (n = 210)	16% (n = 335)
CAUDAL	25% (n = 221)	22% (n = 441)	NOT DONE	NOT DONE

DI (Dorsal interneurons); SLP (Small lateral population).

In the white matter, some ventral edge cells (Fig. 4D–D”, F–F”) and, occasionally, lateral edge cells (not shown) showed glutamate and glycine immunoreactivity in both larval and adult lampreys. All edge cells that express glycine also express glutamate immunoreactivity.

### Fibers

The spinal cord of adult and larval lampreys showed a heterogeneous distribution of glutamate-ir fibers. The highest density of glutamate-ir fibers was observed in the dorsal column (Fig. 2A), except for the caudal spinal region where these fibers were faintly glutamate-ir. The density of glutamate-ir fibers is higher in the dorsal funiculus next to the dorsal column than in the ventral funiculus, although the glutamate-ir fibers were thicker in the ventral funiculus. The glutamate-ir fibers were thicker in adults than in larvae, as reported with respect to the size of the cells. A very rich region of glutamate-ir processes were also observed in the edge cell neuropil. Most of the processes in the lateral neuropil appear to correspond to dense terminal dendritic branches of glutamate-ir edge cells (Fig. 3F–F’). In general, the density of GABA-ir fibers is lower than that of glutamate-ir fibers and the highest density was observed on both sides of the dorsal column and in the margin around the lateral processes of edge cells (as shown with glutamate immunoreactivity), forming a rich GABA-ir marginal neuropil (Fig. 3F–F”). On the other hand, the number of glycine-ir fibers was lower than that of glutamate-ir fibers and they were concentrated in the ventral, ventrolateral and dorsolateral funiculi. However, glycine-ir fibers were thicker than glutamate-ir ones. With respect to the giant axonal system of the spinal cord, the axoplasm of Mauthner and Müller axons did not show glutamate immunoreactivity above background either in larvae or in adults, despite the fact that their perikarya express clearly VGLUT.

## Discussion

Our results demonstrate for the first time the presence of VGLUT mRNA, a highly reliable glutamatergic marker, in numerous neurons of the lamprey spinal cord, thus revealing actual glutamatergic cells. In addition, this is the first comprehensive study reporting the distribution of glutamate immunoreactivity in neurons in the spinal cord of the sea lamprey and the colocalization of glutamate and GABA or glycine immunoreactivities in some spinal cord neurons.

### Glutamate in Spinal Cord Circuits

Excitatory (glutamatergic) cells play an essential role in the CPG networks of the spinal cord. Electrophysiological studies have demonstrated that excitatory spinal interneurons excite both motoneurons and inhibitory premotor interneurons [22]. Present results indicate that these excitatory neurons most probably express a vesicular glutamate transporter, giving support to the opinion that they are actually glutamatergic.

Our results revealed morphological variability among cells of the same type. Although some differences in size can be appreciated among the glutamate-ir cell photographed in a recent study of lamprey [38], these authors focused only on the neurotransmitter phenotypes of commissural cells. A great morphological variability within each type of spinal neurons, including motoneurons, has also been observed in *Xenopus* embryos [58].

Our results indicated that the types of glutamatergic cells were similar in larvae and adults, although their cell sizes were quite different. Another difference between larval and adult lampreys was that the cells were more scattered in the sections in adults than in larvae, as a probable result of the growth of spinal cord volume in adults without accompanying neuronal proliferation. Accommodation of existing neuronal populations to the increasing length and width of the spinal cord between larvae and the adulthood is partially compensated by the great increase in size that takes place in perikarya and their processes.

Our results showed some differences in the glutamatergic populations along the different regions of the spinal cord: rostral, middle and caudal. Specifically, the glutamate-ir dorsolateral cells of the gray matter were observed in the rostral and middle regions but not in the caudal spinal cord. Moreover, the giant glutamatergic cells were only observed in the caudal spinal cord, which is in agreement with anatomical observations on giant interneurons of Rovainen [59] and Selzer [57]. In the following, the main glutamatergic cell populations of the lamprey are discussed and compared with those observed in other species.

### Primary Sensory Neurons (dorsal cells)

Our results showed VGLUT expression in the primary sensory dorsal cells, which is in agreement with electrophysiological studies that indicated that these cells make excitatory monosynaptic and/or polysynaptic contacts with motoneurons [60] and giant relay interneurons [61]. The absence of GABA and glycine immunoreactivity in these cells is also consistent with their excitatory role. It has been proposed that lamprey dorsal cells are homologous to Rohon-Beard cells, which are observed transiently in other fishes and in amphibians during development [59], [62]. Electrophysiological studies have demonstrated that Rohon-Beard cells are glutamatergic in frog tadpoles [63], and they express VGLUTs in larval zebrafish [12].

### Interneurons of the Dorsal Population

A population of glutamatergic cells was observed in the dorsal region of the spinal cord of the sea lamprey. A dorsal domain of cells that express VGLUTs has also been observed in the spinal cord of zebrafish embryos [12]. In lamprey, two types of interneurons can be distinguished in this glutamatergic population: dorsomedial and dorsolateral cells. Dorsomedial cells showed processes directed to the dorsal column region. The dorsal column is constituted by the axons of intraspinal primary sensory neurons (dorsal cells) and spinal ganglion cells that course in the dorsal funiculus to the dorsal column nucleus in the caudal rhombencephalon [64]. Because some cells of the lateral population are excited polysynaptically by dorsal cells, these findings suggest that dorsomedial cells are involved in sensory processing by relaying sensory information [65]. Some cells of this population had a long process that crosses the midline dorsally to the central canal. Cells with similar morphology have been reported in *Lampetra planeri* with classical staining methods [66] and in the sea lamprey using calretinin immunohistochemistry [67]. Cells near the dorsal funiculus that show a dendrite crossing dorsally the spinal midline have also been observed in newt embryos prior to hatching [68]. These cells showed an axon crossing ventrally, which could not be observed in the dorsomedial glutamate-ir cells of the sea lamprey.

The dorsolateral glutamatergic cells were bipolar neurons with a process coursing ventromedially and the other coursing ventrolaterally, but some of these cells showed a branch of the ventrolateral process directed to the dorsal column. This suggests that these cells are also involved in processing sensory information. The ventromedial process of these cells appear to cross the midline ventrally to the central canal in a different transversal level than that occupied by the soma, so they could not be followed to the contralateral side (see Fig. 2C). Their position and cell morphology indicated that these cells are similar to the excitatory dorsolateral commissural cells (dlc) described in *Xenopus* embryos. In *Xenopus*, electrophysiological studies have demonstrated that the dlc cells were excited by the axons of Rohon-Beard cells and in turn, they excite contralateral neurons [69].

### Cells of the Lateral Population

In the sea lamprey spinal cord, a population of glutamatergic cells was observed in the lateral region of the gray matter. During development, the spinal cord of lamprey prolarvae becomes flattened and the cells of the intermediate column migrate away from the midline, giving rise to the lateral gray matter of larvae [34]. Accordingly, this lateral glutamatergic population probably corresponds to the ventral VGLUT2-expressing cell domain of the spinal cord of the embryonic zebrafish [12] and to the VGLUT-expressing neurons of the intermediate region of the rodent spinal cord [3], [26].

Two types of glutamatergic lateral neurons, small and large, were observed in the lamprey spinal cord. Small lateral cells may correspond to interneurons involved in the CPG that excite all types of spinal neurons, including motoneurons, commissural interneurons, inhibitory lateral interneurons and other excitatory interneurons of the ipsilateral side [20], [22]. These lamprey interneurons were considered involved in CPG rhythm generation [25], [70]. With regard to the large glutamatergic neurons observed here, they are probably interneurons since Mahmood et al. [38] did not observe labeled glutamate-ir cells after tracer application to the motor roots of the river lamprey spinal cord. Large lateral cells of lampreys were described as multipolar neurons with dendritic processes directed to the medial, ventral and lateral tracts of fibers, including the Müller and Mauthner axons, which made contacts with them [71], [72]. However, the large glutamatergic interneurons observed here probably do not correspond with these large lateral interneurons studied in lamprey [57], [65], [73], because electrophysiological studies have demonstrated that most of them were inhibitory and only an excitatory lateral cell could be recorded by Rovainen [65]. Our results suggest that large glutamatergic (excitatory) lateral interneurons may be more numerous than expected. Excitatory ipsilateral cells with processes directed to the Mauthner axon and making excitatory synapses on motoneurons, have been observed in goldfish [74].

### Giant Cells

We observed big glutamatergic cells in the caudal spinal cord at the level of the dorsal fin. By their location, size and morphological features, these cells correspond to the giant cells described by Rovainen [59] and Selzer [57]. Electrophysiological studies have demonstrated that these cells are excitatory [59], which is in agreement with the VGLUT expression observed in these cells (present results).

### Cerebrospinal Fluid-contacting Cells

These cells belong to the CSFc rhombencephalic-spinal system observed in all vertebrate species, although they are more numerous in fishes and amphibians (for review see [75]). About the functions of these cells there have been several hypotheses, including among others mechanosensory, chemosensory or secretory functions (see [75]. The recent finding of expression of mRNA of a candidate sour taste sensor (PKD2L1) in spinal CSFc cells of mice [76] supports the hypothesis that they are chemosensory although does not preclude additional functions. In the lamprey spinal cord, some CSFc cells showed VGLUT expression and many were glutamate-ir. The levels of glutamate immunoreactivity varied from faint to strong. All the glutamate-ir CSFc cells were also GABA-ir, which raises the question about the functional significance of this colocalization. GABA-ir CSFc cells have been previously reported in lamprey [29], [32]–[35], [77]. It is worth pointing out that it is the apical dendrite and soma of CSFc cells which primarily showed glutamate and GABA colocalization, whereas only in a few CSFc cells colocalization was observed in the exit of the basal process. The basal (axonal) process of GABA-ir CSFc cells course to the lateral margin of the spinal cord forming a rich marginal plexus [20], [31], making contacts with the dendrites of edge cells [77]. The GABAergic terminals that form the marginal neuropil were glutamate-ir negative, suggesting differential distribution and use by the cell of these neurotransmitters. CSFc cells exert a tonic inhibition on the excitatory edge cells [78].

Differences in the cellular distribution of two neurotransmitters have been observed in other neurons. For instance, rat motoneurons express glutamate in addition to acetylcholine; whereas the endplate at the neuromuscular junction releases only acetylcholine, glutamate is released in the collateral axonal terminals over the Renshaw cells and acts as a cotransmitter [14], [79]. The functions of glutamate in CSFc cells need to be further investigated. In some lamprey CSFc cells, GABA is also colocalized with other neurotransmitters as glycine [31], [53], dopamine [52] and somatostatin [77], suggesting the existence of neurochemically specialized CSFc cell subpopulations.

### Edge Cells

Although lamprey edge cells were first described by Reissner [80], only more than one century later they were characterized physiologically as intraspinal mechanoreceptors [81]. These white matter cells are situated near the lateral edge of the spinal cord and their axons are mainly directed rostrally [24], [65]. Previous electrophysiological results have indicated that edge cells projecting ipsilaterally were excitatory on target cells and presumably glutamatergic, whereas those projecting contralaterally where inhibitory and presumably glycinergic [28], [65]. The existence of glutamate-ir or glycine-ir edge cells has not been mentioned by Mahmood et al. [38], who studied these immunoreactivities in combination with tract-tracing from the contralateral spinal cord. The presence of VGLUT expression observed here in some edge cells is consistent with the physiological characterization of some edge cells as glutamatergic [28]. The observation of glycine immunoreactivity in some edge cells is also in agreement with the characterization of other edge cells as glycinergic inhibitory. Concerning the glycine-ir edge cells, however, a striking result of present experiments is the presence of glutamate immunoreactivity in all these cells, which was observed in both larvae and adults. This fact poses the question about the functional significance of this excitatory amino acid in these edge cells (see below).

### Other Glutamatergic Cells Situated in the White Matter

Numerous small glutamatergic cells situated in the white matter of the spinal cord of the lamprey are associated with the giant Mauthner and Müller axons, and with axons of other reticulospinal cells. These small cells are in a key position to receive descending information from the brain and to participate in locomotion networks. The cells situated in the ventromedial region of the white matter may receive inputs from Müller axons and other reticulospinal axons and may be involved in the locomotion circuits. The association of white matter glutamatergic small cells with giant axons or specific tracts may be complementary to the specific contacts between reticulospinal cells and identified spinal neurons studied by Rovainen [82]. The existence of small glycinergic cells in the white matter receiving contacts from large descending fibers has been also reported previously [30], [31].

### Glutamate-ir Commissural Cells

It seems that there are commissural cells in both dorsal cell populations, but in this study only the cells of which axons decussate at the level of the soma were demonstrated. Excitatory glutamate-ir commissural neurons have been previously described in lamprey using combined immunohistochemical and tract-tracing methods [38], but neither their position in the cord nor their morphology have been studied in detail. Electrophysiological studies have also reported that excitatory contralateral interneurons with descending axons situated in the rostral spinal region excite fin motoneurons in alternating activity with regard to myotomal motoneurons when the lamprey is swimming forwards [83]. However, these authors did no report morphological details that would allow us to compare these cells with the dorsal commissural cell populations described in this study.

### Significance of Colocalization of Glutamate and GABA or Glycine

Double immunofluorescence methods allowed direct comparison of the distribution of glutamate immunoreactivity with those of glycine or GABA in spinal neurons. In several populations of the larval and adult spinal cord, some neurons showed colocalization of glutamate with GABA or glycine immunoreactivities.

As regards colocalization of GABA and glutamate, present results reveal that the cell types that show colocalization in larvae also show colocalization of these neurotransmitters in adults, although some differences were observed in the percentage of glutamate-ir cells showing colocalization. Colocalization of GABA and glutamate in lamprey has been previously reported in some cells of the adult retina [84] and in a few populations of the larval and adult brain [40], [41]. In other vertebrates, colocalization of glutamate and GABA, or of glutamate vesicular transporters and/or GABA synthesizing enzymes or GABA vesicular transporters, has been described in mossy fibers of the hippocampus [85], [86], in retinal cells [87]–[89] and in cerebellar mossy fiber terminals [90]. A striking observation was the colocalization of glutamate and GABA in the body and apical dendrite of some of the CSFc cells, but not in their basal axons, which are only GABA-ir. Release of glutamate and dopamine from different terminals of the retinal bipolar cells has been suggested [89] and release of glutamate and dopamine from different sites of the same cell has been reported in mesoaccumbens projections [91]. Therefore, a similar situation could occur in lamprey CSFc cells containing glutamate and GABA.

The functional implications of glutamate and GABA colocalization are not known yet. It has been suggested that co-release of transmitters could play an important role in improving the precision of the locomotor activity in the spinal cord of mammals [42], [92]. Other functions that have been suggested for this colocalization were its participation in maturing and refinement of synapses [42], [47], [48] and in adaptive processes in the adult brain [90]. However, the functional significance of glutamate and GABA colocalization needs further investigation.

Studies of colocalization of glutamate and glycine are very scarce. In the lamprey spinal cord, colocalization of glutamate and glycine has been previously reported in giant fiber synapses using immunoelectron microscopy [93], although we could not confirm these results. Instead, our study reveals colocalization of glutamate and glycine immunoreactivity in some types of cells of the spinal cord, both in larvae and adults. In other vertebrates, colocalization of glutamate and glycine immunoreactivity has been reported in cells of the retina (human: [94]; chick: [88]) and in vestibular neurons of frog [49]. Colocalization of glutamate and glycine was also observed in vestibular afferents of frog [95], in nerve terminals in the rat locus coeruleus [50] and in axons from the medial nucleus of the trapezoid body in the lateral superior olive [51], [96].

In relation to the functional significance of the glutamate and glycine colocalization, co-release of both neurotransmitters from some central synapses might modulate responses of target neurons. It is known that glycine contributes to excitatory neurotransmission acting as an allosteric modulator for the NMDA receptor [97]–[99]. Moreover, glutamate exerts an allosteric potentiation of the glycine receptor chloride currents. This reciprocal modulation could act as a rapid homeostatic control mechanism for neuronal excitability [99]. A recent study has shown that glutamate co-release during development is crucial for the synaptic reorganization and topographic specification of an inhibitory pathway in the auditory system [51]. Our results showed that glutamate is also present in the glycine-ir (inhibitory) edge cells during both the larval and adult periods. This fact suggests that glutamate and glycine are co-released by some edge cells, but if this is involved in functional modulation of edge cells synapses on target cells needs to be investigated.

### Conclusions

This neurochemical study reports for the first time the presence of a variety of glutamatergic (the same neuronal types were VGLUT positive and glutamate-ir) cell populations distributed along the spinal cord and raises the number of known glutamatergic cell types by two: CSFc cells around the central canal, and white matter interneurons. The high number of glutamate-ir cells in the spinal cord, a feature shared by lampreys and mammals, emphasizes the importance of glutamate in the normal function of the spinal circuits, such as central pattern generator, escape circuits or sensory circuits. In addition, the pattern of VGLUT expression and glutamate immunoreactivity observed in lamprey larvae and adults was similar, indicating that no major changes occurred in this system during metamorphosis. On the other hand, colocalization of glutamate and GABA or glycine has been frequently observed in both larval and adult sea lampreys, indicating that the glutamatergic cells of the lamprey spinal cord are neurochemically more complex than previously thought. The functional implications of such a neurochemical variety of neurons remain to be explored. This work provides a base to study study the function of white matter glutamatergic interneurons, the role that glutamate plays in the CSFc cells and the putative changes in spinal glutamate distribution during spinal cord regeneration in lampreys.

## Materials and Methods

### Ethical Statement

All experiments were approved by the Bioethics Committee at the University of Santiago de Compostela and conformed to the European Union (86/609/EEC) and Spanish (Royal Decree 223/1998) regulations for the care and handling of animals in research.

### Subjects

Large larvae (n = 19∶3 larvae for *in situ* hybridization, 16 for immunohistochemistry; body length between 130–160 mm), downstream migrating young adults (n = 4∶2 for *in situ* hybridization, 2 for immunohistochemistry; body length between 150–170 mm) and upstream migrating adults (n = 6: all of them for immunohistochemistry; length more than 650 mm) of the sea lamprey (*Petromyzon marinus* L.) were used. Larval and young lampreys were collected from the River Ulla (Galicia, NW, Spain), with permission from the Xunta of Galicia. Upstream migrating adults were obtained from a commercial supplier. Larval lampreys were maintained in aerated fresh water aquaria with a bed of river sediment, while adults were processed immediately after arrival to the laboratory.

### Tissue Collection and Processing

Animals were deeply anesthetized with 0.05% benzocaine (Sigma, St. Louis, MO) in fresh water and killed by decapitation. Three spinal cord regions were selected for study. The region between the fourth and the seventh gill is referred to as rostral spinal region, the long region between the end of the gill region and the dorsal fin is referred to as the middle region, and that corresponding to the levels where the dorsal and caudal fins are located is referred to as the caudal region. Pieces of spinal cord for each of the regions (5 mm in length, which corresponds to about 5–6 muscle segments in large larvae and 1 segment in adult lampreys) were fixed by immersion in 5% glutaraldehyde and 1% sodium metabisulfite (MB) in 0.05M Tris buffered saline (TBS; pH 7.4) for 20 h for immunohistochemistry or with paraformaldehyde 4% in phosphate buffered saline (pH 7.4) for 24 hours for *in situ* hybridization. The fixed samples were embedded in Tissue Tek (Sakura, Torrance, CA), frozen in liquid nitrogen-cooled isopentane, sectioned on a cryostat in the transverse plane (14 µm thick) and mounted on Superfrost ® Plus glass slides (Menzel, Braunschweig, Germany).

### *In situ* Hybridization

For VGLUT *in situ* hybridization, we performed the same protocol used previously in studies about glutamatergic cells in the brain of lamprey [40], [41]. The probes used correspond to two sequence fragments (of 584 and 3,483 bp) of a lamprey VGLUT recently cloned in Sylvie Mazan’s laboratory from a lamprey EST database, as reported elsewhere [39]. Plasmid DNA was purified from the selected clones, and the corresponding inserted fragments were excised as control. Templates for in vitro transcription were prepared by PCR amplification from plasmid DNA. A 584-bp 5′ probe was obtained from clone NY0AAA51YH17RM1 using 5′-TTACTGCCGCTGCCAAATC-3′ and the T7 promoter containing sequence 5′-AAGCTCTAATACGACTCACTATAGGGGTAACGCTTGGGCATTCCG-3′ as forward and reverse primers, respectively. A second 3′ 483-bp probe, spanning the region coding for transmembrane domains 9–12, was synthesized using 5′-TGCCCATCGGAGGACAAC-3′ and 5′-AAGCTCTAATACGACTCACTATAGGGGCTCGTCCTCGTTGATGAAG-3′ as forward and reverse primers, respectively. Digoxygenin-labeled riboprobes were synthesized by using the amplified fragments as templates, following standard protocols. In situ hybridizations on cryostat sections (14 µm thick) were conducted using standard protocols with an RNAse A treatment (0.2 µg/ml, 37°C during 30 minutes) that was added to the posthybridization washings. Staining was conducted in BM Purple (Roche, Mannheim, Germany) at 37°C until the signal was clearly visible. The sections were mounted with Mowiol and photographed with a color digital camera in an Olympus photomicroscope.

### Immunofluorescence

For immunofluorescence, sections were pretreated with 0.2% NaBH_4_ in deionized water for 45 minutes at room temperature to quench autofluorescence. Then, sections were incubated with a mixture of a rabbit polyclonal anti-glutamate antibody (Immunosolution, Jesmond, Australia; 1∶4,500) and a mouse monoclonal anti-GABA antibody (Sigma, St. Louis, MO; 1∶1,200) or with a mixture of a rabbit polyclonal anti-glycine antibody (Immunosolution; 1∶3,000) and a mouse monoclonal anti-glutamate antibody (Swant, Bellinzona, Switzerland; 1∶1,000) in TBS with 1% sodium metabisulfite during 3 days at 4°C or overnight at room temperature. After rinsing in TBS, sections were incubated for 1 hour at room temperature with Cy3-conjugated goat anti-rabbit immunoglobulin (Chemicon, Temecula, CA; 1∶200) and fluorescein-conjugated goat anti-mouse immunoglobulin (Chemicon; 1∶100), rinsed in TBS and mounted with Vectashield (Vector, Burlingame, CA).

### Antibodies

The polyclonal anti-glutamate antibody was raised in rabbit against a glutamate-glutaraldehyde-porcine thyroglobin conjugate. The antibody has been tested by the supplier in sections of retina and cerebellum from various mammals and other vertebrates, as well as in dot blot immunoassays with a variety of amino acid-protein conjugates. These include the standard 20 amino acids found in proteins, the non-protein amino acids D-serine, D-alanine and D-aspartate, GABA and the glycine-containing tripeptide glutathione, which did not yield significant cross reactivity. This antibody has been developed by Dr David V. Pow (University of Newcastle, New South Wales, Australia) and used in previous studies of the lamprey brain [40], [41]. In addition, the antibody was tested by Western blotting of lamprey brain/spinal cord protein extracts. This antibody did not stain any sea lamprey brain native protein band in these extracts [40].

The mouse monoclonal anti-glutamate antibody was raised against glutaraldehyde-linked L-glutamate-bovine serum albumin (BSA) conjugate by P. Streit [100], and this clone was made commercially available through Swant. This antibody has been characterized with respect to cross reactivity by antibody dilution experiments as well as by absorption experiments [101]. In addition, the staining pattern obtained with both monoclonal and polyclonal anti-glutamate antibodies in this study and in sections of the brain and retina (unpublished observations) were the same.

The rabbit polyclonal anti-glycine antibody was raised against a glycine-glutaraldehyde-porcine thyroglobin conjugate. It was tested in sections of retina and cerebellum from various mammals and other vertebrates as well as in dot blot immunoassays against a variety of amino acid-carrier protein conjugates, including the standard 20 amino acids found in proteins; the nonprotein amino acids D-serine, D-alanine and D-aspartate; GABA; and the glycine containing peptide glutathione, which did not yield significant reactivity. This antibody has been used in a number of studies of glycinergic neurons of the retina, brain and spinal cord of the sea lamprey [31], [53] and other vertebrates (*Xenopus laevis*: [102]; sturgeon: [103]; zebrafish: [104]; bat: [105]).

The mouse monoclonal anti-GABA antibody was raised against GABA conjugated to BSA with glutaraldehyde and was evaluated by the supplier for activity and specificity by dot blot immunoassay. No cross-reaction was observed with BSA, L-α-aminobutyric acid, L-glutamic acid, L-aspartic acid, glycine, δ-aminovaleric acid, L-threonine, L-glutamine, taurine, putrescine, L-alanine, or carnosine. This antibody showed weak cross-reaction with β-alanine. This antibody has been used in previous studies of the sea lamprey [31], [53], and the pattern of immunostaining reported was the same as in studies with other anti-GABA antibodies [34], [36], [84], [106]–[108]. Moreover, the anti-glycine and the monoclonal anti-GABA antibodies were tested by Western blotting of lamprey brain protein extracts and they did not recognize any brain native protein in blots [31], [84].

### Image Acquisition and Measurements

Immunocytochemically stained sections were photographed and analyzed with a spectral confocal microscope TCS-SP2 (Leica, Wetzlar, Germany). Confocal image stacks were processed with LITE software (Leica). Photographs were adjusted in brightness and contrast with Adobe Photoshop 7 software. To compare the number of cells of each population that showed VGLUT expression or glutamate immunoreactivity, at least thirty sections of two different larvae were counted. In each section, all the cells of each population were counted. Values are expressed as mean ± standard deviation. To measure the cell diameters of glutamate-ir cells, the LITE software was also used. For each neuronal population, at least fifteen cells of two different individuals from each group of animals (larvae and adults) were measured. Values are expressed as mean ± standard deviation. To establish the percentage of colocalization, cells in one out of each four sections were counted in two different individuals from each stage (larvae and adults). Since the small size of cells showing colocalization and due to our aim was not to compare absolute cell numbers or densities in larvae and adults, but the percentage of cells showing neurotransmitter colocalization, no correction factor was used. This need to be bore in mind when comparing percentages in larvae and adults, because the neurons are very different in size.
